# Blockchain-Driven Intelligent Scheme for IoT-Based Public Safety System beyond 5G Networks

**DOI:** 10.3390/s23020969

**Published:** 2023-01-14

**Authors:** Tejal Rathod, Nilesh Kumar Jadav, Sudeep Tanwar, Ravi Sharma, Amr Tolba, Maria Simona Raboaca, Verdes Marina, Wael Said

**Affiliations:** 1 Department of Computer Science and Engineering, Institute of Technology, Nirma University, Ahmedabad 382481, Gujarat, India; 2 Centre for Inter-Disciplinary Research and Innovation, University of Petroleum and Energy Studies, Dehradun 248001, Uttarakhand, India; 3 Computer Science Department, Community College, King Saud University, Riyadh 11437, Saudi Arabia; 4 Doctoral School, University Politehnica of Bucharest, Splaiul Independentei Street No. 313, 060042 Bucharest, Romania; 5 National Research and Development Institute for Cryogenic and Isotopic Technologies—ICSI Rm. Vâlcea, Uzinei Street, No. 4, P.O. Box 7 Râureni, 240050 Râmnicu Vâlcea, Romania; 6 Department of Building Services, Faculty of Civil Engineering and Building Services, Technical University of Gheorghe Asachi, 700050 Iași, Romania; 7 Computer Science Department, Faculty of Computers and Informatics, Zagazig University, Zagazig 44511, Egypt

**Keywords:** blockchain, 6G network, applications, blockchain services, unmanned aerial vehicle, AI

## Abstract

Mobile applications have rapidly grown over the past few decades to offer futuristic applications, such as autonomous vehicles, smart farming, and smart city. Such applications require ubiquitous, real-time, and secure communications to deliver services quickly. Toward this aim, sixth-generation (6G) wireless technology offers superior performance with high reliability, enhanced transmission rate, and low latency. However, managing the resources of the aforementioned applications is highly complex in the precarious network. An adversary can perform various network-related attacks (i.e., data injection or modification) to jeopardize the regular operation of the smart applications. Therefore, incorporating blockchain technology in the smart application can be a prominent solution to tackle security, reliability, and data-sharing privacy concerns. Motivated by the same, we presented a case study on public safety applications that utilizes the essential characteristics of artificial intelligence (AI), blockchain, and a 6G network to handle data integrity attacks on the crime data. The case study is assessed using various performance parameters by considering blockchain scalability, packet drop ratio, and training accuracy. Lastly, we explored different research challenges of adopting blockchain in the 6G wireless network.

## 1. Introduction

In the current scenario, humans interact with the physical world using sixth-generation (6G)-based technologies, such as the Internet of things (IoT), cloud computing, big data, and artificial intelligence (AI). These technologies monitor the application’s resources and control a colossal amount of data, power, and storage resources. The advent of IoT technologies brings tremendous cognizant applications, such as smart healthcare, smart cities, smart grids, etc., to improve people’s standard of living. However, the increasing number of users and their network demands, such as high bandwidth and low latency, make wireless communication networks more complex and vulnerable to different security and privacy issues, such as user identity, authenticity, data integrity, information security, and confidentiality [1]. For example, in a smart healthcare system, the patient health record is private and sensitive information that must be secured from adversaries, which can misuse this information by tampering with it or selling it to the black market. Another example is for public safety applications (e.g., IoT-based crime detection), where a criminal tries to modify the central repository that holds the criminal records. The adversary can modify or tamper the criminal records, i.e., it can perform injection attacks to change the criminal’s identity (e.g., remove the traces of criminal evidence). Therefore, superior technology such as blockchain is required to overcome the aforementioned security issues in 6G-based smart applications [2]. It strengthens the security and privacy of wireless communication networks by utilizing its essential characteristics, i.e., immutability, scalability, security and privacy, transparency, and decentralization [3,4].

Blockchain is a decentralized ledger technology that builds trust between various network services and applications such as smart grids, smart agriculture, smart cities, unmanned aerial vehicle (UAV) communications, and vehicle-to-vehicle (V2V) communications. It also delivers improved services such as interoperability, distribution, immutability, faster settlement, unanimity, and many more. As a result, blockchain is associated with a 6G-based wireless communication network and offers a secure and trusted environment for data protection in an IoT-based public safety environment. For example, Velliangiri et al. [5] discussed the applicability of blockchain technology with the emerging 6G network for secure data sharing. They proposed a privacy-preserving architecture for industrial services and applications. Then, Bindu et al. [6] discussed the significance of blockchain in 6G wireless communication networks. They studied various applications of 6G with security and privacy threats and explored blockchain-based solutions for smart and industrial IoT-based applications. In [7], the authors used blockchain secure storage to store crime images by applying watermarking and cryptographic blockchain in forensic applications. There has been a lot of research work in the field of the integration of blockchain in 6G-based real-world applications. A detailed explanation of each application is as follows.

### 1.1. Applications of Blockchain in 6G Network

**Figure 1:**
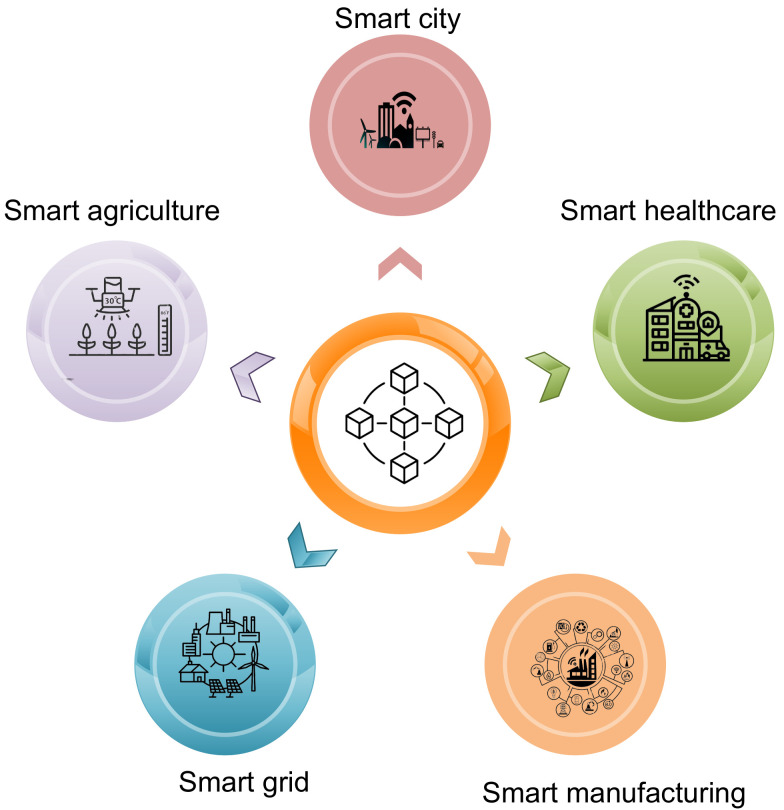
Applications of blockchain in 6G network.

This section presents the role of blockchain in 6G-based smart applications such as smart cities, smart manufacturing, smart agriculture, V2V communication, UAV communications, and smart grid (Figure 1).

#### 1.1.1. Smart Healthcare

The healthcare industry is an essential sector in the nation’s economic system that offers medical services to treat patients, promote patients’ health, and increase the quality of life of individuals. The emerging 6G technology revolutionizes healthcare by changing the perception of lifestyle [8]. A smart healthcare system offers better connectivity between patients and healthcare providers, medical organizations, and IoT-based medical devices. It provides a health information platform by adopting 6G-based services, such as augmented and virtual reality. In addition, healthcare systems require the blockchain to securely store medical records and update patient data across different platforms in real-time [9,10].

Researchers enabled blockchain technology in 6G-based applications and proposed many solutions for smart healthcare. For example, during the COVID-19 pandemic, patients suffering from chronic diseases are more at risk than ordinary people because patients with diabetes and asthma are more susceptible to coronavirus infection. Therefore, there is a requirement to treat these types of patients remotely and offer them emergency facilities such as temperature checks, pulse, and respiratory and O${}_{2}$ monitoring. However, remote diagnosis and treatment can be lured by the attacker and cause patient data security issues. Thus, to overcome these issues, blockchain is integrated with a healthcare system that remotely monitors the health data of patients suffering from a chronic disease. In [11], the authors facilitated a private blockchain and a solidity-based smart contract that keeps track of patients’ health information. The role of blockchain in their proposed work is to securely analyze patients’ details and store patient health records from and to the sensors. Further, in [12], the authors investigated AI with healthcare systems and proposed a parallel gout diagnosis and treatment system (PGDTS). Their proposed system enhances the diagnosis accuracy and the effectiveness of medical treatment. After the diagnosis and treatment process, their adopted scheme was analyzed and evaluated based on diverse therapeutic regimens, and they established parallel execution for real-time optimization of healthcare processes. Their integrated blockchain-based PGDTS scheme offers integrity, scalability, and security of patients’ data.

#### 1.1.2. Smart Manufacturing

In the real-world scenario, massive amounts of data are exchanged between the devices for large-scale manufacturing. Therefore, machines have the interaction capability to cooperate via the Industrial Internet in the manufacturing industry. In order to improve the performance of the entire manufacturing process, machines autonomously make local decisions [13]; however, there is a requirement for always-on connectivity, a higher data rate, more reliability, and ultra-low latency in the manufacturing industry. The 6G mobile communication techniques are developed to target the aforementioned manufacturing industrial services; however, there are security issues in the manufacturing industry where a malicious user could tamper with the manufacturing data. Therefore, blockchain-based networks are integrated into manufacturing systems that offer cybersecurity guarantees via smart contracts, hash functions, digital signatures, and immutable digital ledgers to tackle security and privacy issues. Recently, Leng et al. [14] incorporated digital twins with decentralized permissioned blockchain networks to build better mutual trust and data flow in manufacturing processes. They proposed a ManuChain prototype to realize lower- and upper-level intelligence for crowd self-organize and holistic optimization. Their proposed scheme aided a new value for transforming and upgrading manufacturing systems. It also enhanced the efficiency of the manufacturing process.

Recently, blockchain has been investigated and integrated with mobile edge computing (MEC) to overcome the computational workload. It reduces time complexity and significantly improves system performance. In [15], the authors presented a smart manufacturing system by integrating MEC with blockchain technology. They also proposed a swarm-intelligence-based optimization model with numerous tasks that enhance the processing time. Blockchain offers device-level information transmission and manufacturing service transactions in their proposed approach. Then, Teng et al. [16] proposed a smart manufacturing scheme using distributed ledger technology that supports on-demand production. They introduced a blockchain-embedded smart manufacturing system that enhances manufacturing efficiency. They investigated latency and limited throughput issues in the blockchain network. Therefore, they proposed a deep Q-network that maximizes the user’s net profit. Their results show that their proposed framework is highly scalable. It offers consistency, availability, and privacy by incorporating off-chain storage, ledger isolation, and encryption mechanism.

#### 1.1.3. Smart City

Smart city architecture requires conventional internet support for information collection and transmission; however, the transmitted data encounter latency and security issues due to the precarious communication link between nodes of the smart city. Therefore, 6G communication technology plays an essential role in connecting devices such as the sensor in the IoT environment. However, these devices face authentication issues that affect computational costs, communication overheads, and robustness [17]. To mitigate these issues, blockchain technology offers confidentiality, integrity, and availability in IoT-enabled smart devices.

Several researchers discussed the security challenges in a smart city context. In [18], the author aimed to enhance the data collection and processing in smart city governance. Therefore, they introduced a hybrid consortium blockchain framework that encourages public engagement in the decision-making process. The proposed approach can tackle the collusion attacks from the public verifier’s group. Further, they offered a Stackelberg-game-based approach to enable persistent public participation in the transaction validation process. The work in [19] proposed an IoT–fog–cloud integrated approach with the interplanetary file system (IPFS)-based blockchain for IoT-driven smart cities. They presented a secure framework based on two-level privacy and an intrusion detection scheme. The first scheme incorporated a blockchain module to secure the IoT information and proposed a dimensionality reduction technique that transits raw IoT data in a new shape. They applied a gradient boosting anomaly detector approach for intrusion detection. Their results show that their adopted scheme outperforms in terms of accuracy, recall, F1-score, and precision compared with the conventional intrusion detection scheme. Further, Rahman et al. [20] incorporated blockchain with the IoT infrastructure and proposed an MEC-enabled sharing economy system for mega smart cities. They utilized smart contracts for decentralized messaging services and stored IoT data in a decentralized repository via edge networks.

#### 1.1.4. Smart Vehicles

To establish V2V communication, vehicles must quickly send the vehicular information, comprising number of pedestrians, road conditions, number of vehicles, and their conditions, to reduce the number of road accidents and enhance road safety. However, the scalability of V2V communication increases the security, safety, reliability, and trust issues of vehicular network users. Therefore, there is a requirement for a prominent solution to overcome these issues. Blockchain is used to manage the ground truth of vehicle information and offers data authentication among vehicles. Many researchers recently focused on blockchain technology for V2V communication. Chattaraj et al. [21] integrated blockchain with IoT-driven smart devices that improve customer safety, ease congestion, and pollution reduction. For that, they proposed a certificateless key agreement protocol for Internet of vehicles in a smart transportation context (Block-CLAP). The proposed scheme outperforms the precedent authentication schemes’ security, communication, and computational overheads.

Furthermore, Xia et al. [22] integrated blockchain with the Internet of vehicles (BIoV) and proposed a novel V2V electricity trading approach using Bayesian game pricing. They utilized a Bayesian game that predicted the price in the distributed BIoV with incomplete information-sharing. In the pricing game approach, they applied smart contracts that provide security, trust, and reliability for electricity transactions. The results reveal that the proposed scheme reduces communication overhead and time complexity in the decentralized IoVs compared to the static game. Then, in [23], the authors incorporated a blockchain network with the vehicles that stores real-time information about vehicles, such as relaying, transmitting, and receiving data. As a result, the adopted scheme is able to block malicious or unauthorized vehicles from participation. Finally, they considered network parameters, such as throughput and error rate analysis (i.e., bit error rate) to evaluate their proposed scheme. They proved the proposed scheme’s efficiency and effectiveness by enhancing throughput by 3 bps and decreasing BER by 2 dB.

#### 1.1.5. Smart Grid

A smart grid is introduced to meet people’s higher electricity needs and optimize resource allocation. The smart grid makes better decisions about power and energy-sharing data. It supports management approaches for peak energy demand and also reduces energy consumption by exchanging the data of energy feedback and power consumption [24]. However, due to the possibility of malicious access during information exchange over the insecure communication network, multiple entities are unwilling to convey their information to protect their data privacy. Moreover, there are various types of cyberattacks in smart grid systems, such as session hijacking, nonrepudiation, denial-of-service (DoS), flooding, spoofing, and man-in-the-middle (MiTM) attack [25]. To overcome the security and privacy issues, smart technology is required that provides trust, reliability, and data-sharing security. Many researchers adopt blockchain as a solution to secure the smart grid. For instance, the authors of [26] presented a secure and reliable data exchange scheme by utilizing the processing mode. The proposed scheme provides transparency and auditability of energy production, pricing, and services in smart grids. They utilized smart contracts to authenticate and track the data flow between different blockchain members. Additionally, they incorporated a distributed ledger that provides an immutable and transparent data usage record. As a result, their adopted scheme outperforms in terms of payoffs compared with baseline works. Further, Hao et al. [27] focused on the efficiency and privacy issue in the current electricity trading scheme. Therefore, they analyzed a decentralized scheme of peer-to-peer electricity trading by introducing smart meters. They utilized a blockchain wallet for transactions and a smart contract to initiate the transmission switch instructions. The results revealed that the proposed scheme reduces the cost and latency. Furthermore, they offered fast and privacy-aware roaming to prove the proposed scheme’s feasibility and efficiency.

#### 1.1.6. Smart Agriculture

In the world, agriculture is the largest sector of economic activity. With an increasing world population, there is a requirement to increase agricultural productivity by adopting smart technologies, such as IoT, robotics, UAV communication, AI, etc., to enhance the quality and quantity of agricultural products with minimum human labor. Existing wireless standards such as ZigBee, Bluetooth, Wireless Fidelity (Wi-Fi), Narrowband IoT (NB-IoT), and WiMAX are used for pesticide detection, soil sensing, smart irrigation, crop recommendations, and plant protection. They prevent forest fires and offer safety to crops from pests [28]. However, the aforementioned technologies face an issue when a considerable amount of agricultural data is accumulated in real-time. In addition, population growth and environmental effects such as climate change, temperature, rainfall, etc., affect the agriculture system. Therefore, emerging 6G wireless communication technology and its specific application scenarios are introduced in agriculture, providing intelligent, unmanned, and automatic agricultural production and cultivation by offering high-speed connectivity between smart devices.

Smart agriculture systems require transparency and commercial security for the food data [29]. Therefore, many researchers adopt blockchain technology in the agricultural field to provide security, reliability, transparency, and privacy of agricultural data. For example, Vangala et al. [30] proposed a blockchain-based smart contract that includes a key agreement approach to mitigate the risk of data integrity attacks in smart farming systems. Their proposed scheme utilizes a robust authentication method between two IoT devices. Similarly, another authentication phase is utilized between an IoT device and the gateway node to offer endogenous security. Furthermore, they proposed a hybrid blockchain to store the trading transactions between different components of the agriculture ecosystem, pesticide usage, and quality and quantity of fertilizers. The results revealed that the adopted scheme outperforms in terms of computational time compared to the existing authentication protocols. Then, Yang et al. [31] focused on centralized, opaque, and untrustworthy data storage issues for horticulture products. To overcome the aforementioned issues, they introduced a traceability system (e.g., on- and off-chain traceability) using a dual storage within the blockchain. They utilized cryptography-based elliptic curves with blockchain for safe data sharing. Moreover, they proposed a reputation-based smart contract to upload traceability information from the node. Their simulation results enhance efficiency and guarantee the agriculture data’s reliability, security, and authenticity.

Nevertheless, the aforesaid applications require superlative care from criminal activities, natural calamities (e.g., fire, earthquake, etc.), terrorist attacks, and riots. For example, if a person driving a smart vehicle meets with a road accident, it can use V2V communication to share this information with the emergency medical response team. Similarly, UAV communication can assist V2V communication in sharing this information with nearby hospitals to provide emergency medical care. Another scenario is where smart manufacturing requires on-site maintenance to clean and repair various critical equipment of the manufacturing organization. This is challenging for a person to perform on-site maintenance because the manufacturing vessels and equipment are highly hazardous (i.e., radioactive) to human health. Further, a criminal attempt to commit a crime (e.g., stealing, murder, kidnapping, etc.) in the smart city must be captured under the jurisdiction. Therefore, the government and local organizations must offer an emergency response, disaster risk management, and criminal investigation teams under the umbrella of public safety applications to protect the wellbeing of the nation’s citizens who are using the abovementioned applications. However, it is observed from the literature review that public safety applications are highly insecure from the perspective of security and privacy. These applications still use legacy software and tools and utilize a centralized repository for data storage that the attackers can lure. Moreover, as these applications are mission-critical, they require low latency and high data rate communication to offer proactive services; however, many public safety applications still use traditional network interfaces (e.g., 3G and 4G) that degrade their performance.

### 1.2. Motivation

The motivation behind this work is given as follows.

- Most of the existing literature focuses on the role of blockchain in 6G-based applications; however, there are a few research articles available in which the authors studied the drivers, enablers, security requirements, and applicability of blockchain in 6G-based smart applications.
- From the literature review, we observed that no comprehensive work exists that considers the security and privacy challenges of IoT-based public safety applications using blockchain technology.
- Thus, there is a stringent requirement to provide exhaustive work on the applicability of blockchain in IoT-based public safety applications underlying a 6G network.

### 1.3. Research Contributions

This article presents the role of the blockchain in a 6G-based IoT-enabled public safety application. We also emphasize how blockchain provides security and privacy in 6G services. Moreover, the paper shows the amalgamation of blockchain in 6G communication networks to strengthen the security and communication of public safety applications. Based on that, the following are the significant contributions of this research article.

- Through this article, we propose a detailed work on the applicability of blockchain in a 6G network. We also analyze the 6G-based smart applications and discuss how blockchain features provide trust, security, authenticity, confidentiality, and privacy.
- We explore different 6G-network-based services and discuss the influence of blockchain in delivering 6G-based services to various smart applications.
- We consider a blockchain- and 6G-based case study for public safety applications to showcase the adoption of blockchain technology in the 6G network interface. Further, the case study is assessed by considering blockchain scalability, 6G latency, and AI statistical measures parameters.
- Lastly, we perform an in-depth analysis and identify the research challenges of the adoption of blockchain in the 6G network to motivate the researchers working on the same domain.

## 2. Related Works

In the previous section, we discussed various blockchain-based 6G applications such as smart healthcare, smart grid, smart agriculture, smart manufacturing, etc. After studying these real-world applications in detail, we identified that public safety is an essential domain that requires more security attention. Various researchers consider public safety applications and offered security and privacy approaches for road safety, criminal activity monitoring, on-site safety, etc. They deal with data security, person authentication, information confidentiality, and integrity issues. For example, Ihinosen et al. [32] addressed the dynamic ridesharing issue in public transport systems. They proposed GPS tracking and a two-way rating system using the mobile application’s facial recognition module.

In the aforementioned work, the researchers incorporated different security mechanisms using mobile applications and cryptographic primitives. However, they are yet to consider data consistency, traceability, and scalability. Therefore, Xu et al. [33] utilized various technologies such as blockchain, IoT, edge, fog, and cloud computing for smart public safety. They proposed the BLockchain-ENabled Decentralized Microservices Architecture for Smart public safety, named BlendMAS, scheme that offers security and data access control for the public safety system. Then, in [34], Fitwi et al. worked on the safety and security of video surveillance systems. They proposed edge computing and a smart-contract-based lightweight privacy protection (Lib-Pri) scheme that identifies the fugitives using their facial features. Makhdoom et al. [35] proposed a smart-contract-based collaborative intrusion detection system that prevents cyberattack and data theft. Their proposed scheme manages the trust between the nodes and prevents the input false attack in the IDS.

Furthermore, Wu et al. [36] focused on the on-site safety issue due to the equipment defects and environmental hazards for tower crane safety management. They proposed a blockchain-based framework utilizing Hyperledger Fabric that offers security and authenticity with trust at the construction site. Moreover, Yu et al. [37] addressed data vulnerability, confidentiality, privacy, and the centralized key storage issue of the IIoT data storage applications. To overcome this problem, they proposed a Shamir threshold cryptography scheme with blockchain called STCChain that offers availability, accountability, and integrity of the data. Then, Na et al. [38] proposed a blockchain-enabled access control scheme using multisignature that protects autonomous vehicle data. They collected and stored vehicle data using a dashboard camera and global positioning system. Their findings showed that the proposed scheme offers data privacy and security with lower verification time and latency. Table 1 shows the comparative analysis of existing works for public safety applications by incorporating security, 6G network with their objective, pros, and cons. From the overall study, we observed that many researchers carried out a lot of research work in the security field for smart public safety applications. In most papers, the research community focuses on the role of different security approaches, such as cryptographic and mobile applications. However, very few of them incorporated blockchain-based security in public safety applications. Therefore, we included a blockchain-based security approach using 6G wireless communication in the public safety application domain.

### Organization

The rest of the research article is organized as follows. Section 2 presents the literature review of the state-of-the-art works. Section 3 presents blockchain-based 6G services such as network slicing, spectrum sharing, data sharing, and resource management. In Section 4, we proposed layer-wise architecture for IoT-based public safety application. Section 5 discusses the results and analysis of the proposed case study. Then, we discuss various challenges of the adoption of blockchain in 6G network in Section 6. Finally, Section 7 provides the conclusion.

## 3. Role of Blockchain in 6G Services

This section provides insights on the 6G-based smart services, such as network slicing, spectrum sharing, resource management, and data sharing. Table 2 shows the comparative study on blockchain-based existing 6G services.

### 3.1. Network Slicing

Network slicing allows to create different virtual networks, where each network poses different network services from the physical networks infrastructure. This is made possible by integrating technologies, such as software-defined network (SDN) and network functions virtualization (NFV), that divide single network connections into multiple distinct virtual connections. It delivers virtual applications that are directly deployed on the slice for instant service delivery to the users. As an outcome, mobile applications that are running on slices have large-scale connections and low-latency services with a high-quality user experience. Network slices reduce the network complexity by virtualization and add automation to a complex network. However, it faces issues in dynamic slice creation, resource sharing, wireless resource virtualization, and data security. To mitigate the aforementioned issues, blockchain is integrated with network slicing to provide user trust, transparency, and privacy. Many researchers integrate blockchain as a service in network slicing. For example, Abdulqadder et al. [39] focused on user authentication, load imbalance, and inappropriate network slicing issues in an edge-assisted 6G network. Initially, they designed a deep-learning-based adversarial network that predicts the efficient network slices and communication links based on the slice capacity, priority, and quality-of-service (QoS) demands for data transmission. Further, they applied DAG-based blockchain using the proof-of-space approach to enhance the scalability and overcome more resource consumption issues. Moreover, they introduced a soft actor–critic scheme for load balancing in the SDN controller. Finally, the paper concluded that the adopted scheme reduces handover latency, packet loss rate, and data transmission delay and enhances the system throughput and different attack detection rate.

He et al. in [40] integrated a multidomain network slice orchestration framework with a blockchain-based CoNet consensus algorithm for data consistency, scalability, and security. Further, they proposed a game-theory-based bilateral evaluation approach that provides system fairness with a QoS guarantee. The findings showed that the adopted scheme provides users’ data security and privacy with high throughput and low latency. Then, Chen et al. [41] formulated a blockchain-based optical network slicing scheme for IoT applications. They focused on the users’ data security and trust issues while they utilized IoT services. The results showed that their presented scheme is able to complete slicing transactions with a high-quality dedicated communication network.

### 3.2. Spectrum Sharing

The explosive growth of bandwidth-intensive Internet applications such as live audio and video streaming, web conferencing, big data processing, etc., requires high-speed network access with a higher frequency spectrum. The 6G wireless technology provides high-speed network access with ultra-reliable low-latency communication (URLLC) using a higher-frequency spectrum [42]. However, it also increases congestion and interference in the communication network. To overcome this issue, it is required to share the spectrum properly that enhances communication capacity and spectrum efficiency. Spectrum sharing aims to efficiently use scarce natural resources to ensure fairness between mobile users. There are two types of methods for spectrum sharing: static and dynamic spectrum sharing. Out of these two spectrum-sharing mechanisms, dynamic spectrum sharing is the most adopted method; it provides feasible, flexible, and efficient spectrum management based on cognitive radio technology [43,44]. The variety of spectrum bands and the rivalry between different mobile network operators are the challenges in the 6G spectrum sharing. After lots of research, many solutions were proposed in [45,46] for efficient spectrum sharing; however, the solutions that address the spectrum scarcity problem face issues such as single point of failure, malicious activity from unauthorized users, and security concerns.

To mitigate the aforementioned issue, many researchers incorporate blockchain with 6G technology, which improves security and system performance [47]. For example, Liu et al. [48] integrated AI with blockchain for radio spectrum resource allocation in 6G-based enhanced URLLC service. They focused on resource scheduling, classification, and neural network optimization issues between IoT devices. For that, they proposed a reinforcement learning approach with a blockchain-integrated hybrid cloud. Their presented scheme uses unified coding and identification standards to build a public management platform to register and manage IoT device information. The findings reveal that the adopted scheme effectively increases spectrum utilization with security and reliability. Further, in [49], the authors presented a blockchain-based user-autonomy spectrum-sharing framework that efficiently manages the heterogeneous devices of the IoT network. They designed a swarm intelligence approach and a dynamic tip selection algorithm to enhance the system and global utility. The simulation results revealed that the proposed scheme minimizes the average waiting time of transactions and power consumption. Moreover, it enhances global utility, throughput, and spectral efficiency. Then, Manogaran et al. [50] presented a blockchain-based security solution for access control and preserving the data privacy of users’ resources. Furthermore, they introduced a Q-learning approach with machine-type communication (MTC) that enhanced the performance in the 6G environment. Compared to the existing schemes, their proposed scheme outperformed in terms of the success ratio, access time modification, time complexity, and memory utilization.

### 3.3. Data Sharing

The 6G-based smart applications such as UAV communication, aerial base stations, satellite communication, V2V communication, etc., offer real-time data-sharing features over the Internet through a mobile communication network. Moreover, an increasing number of social media applications, such as Twitter, Facebook, Instagram, etc., and web conferencing applications, such as Webex, Google Meet, Zoom, etc., require content delivery in real-time. These applications generate a colossal amount of audio, video, text, and image data. To control and share a large scale of data requires an efficient data-sharing scheme that overcomes data leakage and security threats. Decentralized blockchain technology offers traceability, security, privacy, tamper-resistance, transparency, and immutability to handle data-sharing issues in the 6G network. Khowaja et al., in [51], aimed to offer a secure and reliable data-exchange scheme using a private blockchain, i.e., Hyperledger Fabric for vehicular networks. Additionally, they developed a deep-learning-based autoencoder segmentation approach that gathers the vehicles based on their similarity. The proposed data-sharing scheme achieved better throughput, CPU utilization, efficiency, applicability, computational overhead, and time complexity.

Further, Zhang et al. [52] combined permissioned blockchain with federated learning (FL) and proposed a channel-based distributed data-sharing scheme for data sharing and preserving the privacy of the user. Moreover, they proposed a data storage scheme using on- and off-chain data storage for authenticating identity, data, and transactions. The adopted scheme enhanced data sharing and security, improved data model quality, and prevented malicious attacks. Then, in [53], the authors proposed a concrete access control mechanism for vehicular communication (vehicular ad hoc network (VANET)). For that, they integrated blockchain with ciphertext-enabled attribute encryption (CP-ABE) for user and data storage identity management. Further, they enhanced the security mechanism and designed the advanced attributed algorithm that minimizes the lightweight device’s computational pressure in VANET. The findings show that the proposed scheme provides effective data security with low performance overhead.

### 3.4. Resource Management

The most important service in a 6G wireless communication system is utilizing various mobile resources such as memory, bandwidth, power, and channel. However, managing these resources and fulfilling user demands, such as on-demand service and real-time visibility, is critical. Moreover, an increasing number of smart applications, such as smart cities, smart homes, e-healthcare, smart grid, smart agriculture, autonomous vehicle, holographic communication, etc., require real-time access to various resources. Therefore, resource utilization, allocation, and management are required based on user needs. Many researchers have proposed efficient and optimal resource allocation schemes [54,55,56,57]; however, these schemes are centralized, where a single authority verifies the user and allocates resources as per their need. They face various security threats, such as user identity, information confidentiality, malicious attacks, and a single point of failure. Blockchain is considered to be a promising technology to address the abovementioned issues. For example, Li et al., in [58], integrated blockchain with MEC-based IoT applications to enhance the performance of network attributes and ensure data-sharing authenticity. They presented the cloud–edge collaborative resource allocation mechanism by introducing a collective reinforcement learning (CRL) scheme. The authors jointly optimized the data offloading, blockchain node intervals, and transmit power to enhance the energy efficiency of the proposed scheme.

In [59], the authors incorporated blockchain technology with the Internet of Everything (IoE) applications. They proposed a blockchain-enabled metaheuristic approach for resource allocation in a cyber twin-driven 6G-IoE environment. Additionally, they designed an optimal resource allocation algorithm using graph clustering based on a quasi-oppositional search and rescue optimization approach. Their findings showed that the proposed work reduced average system cost to 2.63 and power consumption to 0.012 mW compared to the predefined work. Further, Yang et al. [60] intended to optimize the Industrial IoT device’s energy allocation by reducing energy consumption and computation overhead. Therefore, they incorporated blockchain with IIoT systems and proposed a deep Q-learning (DQL)-enabled optimization framework that ensures data security and energy efficiency. Finally, the proposed scheme is compared with existing approaches, and it performs better in terms of the computation overhead and weighted cost.

**Table 2:** A comparative analysis of blockchain-based 6G services.

	Author	Year	6G Application	Objective	Implication of Blockchain	Methodology	Remarks
Network slicing	Abdulqadder et al. [39]	2022	NFV and SDN	Context-aware authentication handover.	The proposed scheme tackle security, QoS guarantee, and improper resource utilization challenges through network slicing and load balancing.	Generative adversarial network and DAG-blockchain.	Not focused on the privacy threats of the 6G.
	He et al. [40]	2021	NFV and SDN	Multi domain network slicing.	Offers end-to-end network slice orchestration services and privacy-preserving scheme for private network.	CoNet consensus algorithm.	The scheme does not consider time complexity for multiparty computation.
	Chen et al. [41]	2020	IoT	Optical network slices for user.	To provide user’s data security and trust.	Blockchain-based optical network slicing approach.	Does not consider other performance evaluation parameters such as throughput, scalability.
Spectrum sharing	Liu et al. [48]	2021	IoT, cloud	Radio spectrum resource sharing -tructure in eURLLC.	Integrate blockchain with hybrid cloud to register and manage the information of IoT devices.	Reinforcement learning.	Does not discuss energy efficiency.
	Zhang et al. [49]	2021	IoT	To manage a large-scale IoT network with heterogeneous devices.	DAG-blockchain for user-autonomy spectrum sharing.	A dynamic tip selection and swarm intelligence method.	Focused on the unlicensed bands.
	Manogaran Manogaran et al. [50]	2020	MTC	Secure reliable service delegation in 6G.	Incorporates blockchain with security measure that provide access control, security, and privacy-preserving for the resources and the users.	Q-learning.	The proposed scheme focused on the virtual resource sharing.
Data sharing	Khowaja et al. [51]	2022	VSN	Efficient and secure data sharing.	The scheme proposed Hyperledger Fabric for data-sharing security.	Stacked autoencoders and density-based clustering method.	The presented scheme is not able to handle broadcasting security issues.
	Zhang et al. [52]	2021	FL	State-channel-based distributed data sharing for sandbox.	They proposed permissioned blockchain with FL for data sharing.	Fine-grained data access control model.	Does not take time complexity and computation overhead.
	Li et al. [53]	2020	VANET	Distributed data storage for vehicles and fine-grained access for VANET data.	They integrated blockchain with ciphertext-based attribute encryption.	HECP-ABE algorithm.	Does not consider data security in term of level of anonymity and stateless access.
Resource management	Li et al. [58]	2022	MEC and IoT	Intelligent resource allocation.	They incorporate practical Byzantine fault tolerance protocol for the data privacy.	Collective reinforcement learning.	Does not consider the user’s privacy and task offloading scenario when the user is outside of the covered area.
	Jain et al. [59]	2021	IoE	Optimal resource allocation.	Introduced blockchain for system’s monitoring, assuring safety, managing, and sharing resources effectively.	Metaheuristic with blockchain.	The proposed scheme does not talk about the computation overhead of the system.
	Yang et al. [60]	2020	MEC and IIoT	To optimize the IIoT device’s energy allocation.	They combined blockchain with MEC to solve the joint optimization problem.	Deep Q-learning.	They do not focus on the network access scenario when large-scale devices are connected.

## 4. Case Study: Blockchain, 6G, and UAV-Based Collaborative Architecture for Public Safety Application

**Figure 2:**
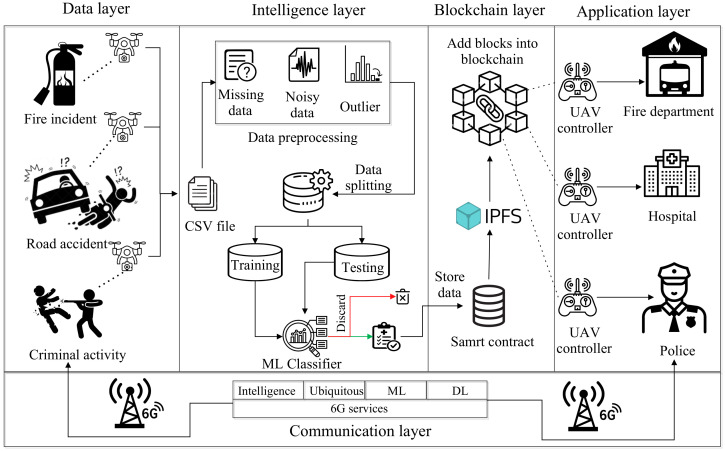
Proposed architecture.

This section shows the working of the proposed architecture, which is AI-, blockchain-, and 6G-based secure UAV communication for public safety scenarios such as fire incidents, road accidents, and criminal activity. Figure 2 shows five layers of the proposed architecture, i.e., data, intelligence, blockchain, application, and communication layers. The working of each layer is discussed as follows.

### 4.1. Data Layer

In this layer, we considered different emergency services, such as fire incidents, road accidents, and criminal activity, which require faster communication to deliver sensitive information (e.g., road accidents and medical emergencies) to the entities of the application layers. Here, UAVs are incorporated to monitor the abovementioned scenarios and collect data from them to assist in making a decisive decision for different public safety applications. For instance, if a criminal attempts to perform a crime at a particular location, the UAVs collect the data from the location and forward it to the nearby police station. However, the criminal can hire an attacker to perform adversarial attacks on the collected data to avoid legal protection. An attacker can perform different network-related attacks to exploit the crime data stored in the central repository of police stations. Therefore, there is a requirement for an intelligent and automated approach that can seamlessly detect nefarious patterns in the network and eradicate it from the public safety environment.

### 4.2. Intelligence Layer

In this layer, we apply different machine learning (ML) classifiers that classify the data into attack and nonattack categories. As discussed in the data layer, UAVs collect data from various application scenarios and store that data in comma-separated value (CSV) files. The feature space of the dataset is discussed as follows.

#### 4.2.1. Dataset Description

In that direction, first, we collect the criminal activity dataset from the Kaggle [61] that classifies the data into attack and nonattack from the criminal’s behavior. However, the collected dataset is not enough to identify the attack or nonattack category; therefore, we accumulate more data from different sources having relevant criminal activity information. The final dataset Ψ consists of different attributes such as location, crime type, weapon type, a weapon used or not, etc. It has *n* rows, $a_{1},a_{2},a_{3},\ldots ,a_{n}$ and m columns, $b_{1},b_{2},b_{3},\ldots ,b_{m}$.

(1) $$\begin{array}{c} a_{1},a_{2},a_{3},\ldots ,a_{n}\in \Psi \end{array}$$

(2) $$\begin{array}{c} b_{1},b_{2},b_{3},\ldots ,b_{n}\in \Psi \end{array}$$

where *n* is the total count of row 1575 and *m* is the total count of column 9. The collected dataset is stored in the comma-separated values (CSV) file and further passed to the next layer, which is the intelligence layer. Here, there is a risk of data modification on the judicial data collected from the aforementioned critical application scenarios. Therefore, there is a need for data security that protects sensitive data from malicious threats.

#### 4.2.2. ML Classification Approach

The CSV file has missing values, noise, and unmanaged data columns that affect the performance of the ML classifier. To overcome this issue, we replace the missing value with central tendency, i.e., mean value. Further, the dataset has numeric and textual values (alphanumeric), such as location, crime, and weapon types; therefore, there is a need to convert nonnumerical data into numeric data. We apply the label-encoding technique $L_{e}$ that assigns a numerical value to each categorical value.

(3) $$\begin{array}{c} \forall b_{3},b_{4},b_{5}\overset{\mathrm{apply}}{\to }L_{e}\in \Psi \end{array}$$

After reading the CSV file, we realize that the dataset attributes have different datatype *D*, such as an object, int, and float. However, certain ML classifiers do not recognize attributes with object datatype. To overcome this issue, we change the datatype from floating to the int using the astype() function.

(4) $$\begin{array}{c} \forall b_{7},b_{8}\overset{\mathrm{change}}{\to }D \end{array}$$

Then, we analyze outliers that corrupt the dataset and mislead the ML classifiers for the wrong classification. For that, we apply outlier detection techniques that normalize the data using a standard scale without distorting differences in the ranges of values or losing information. Various normalization approaches exist, such as min-max, standardization scaling, and Z-score. Here, we apply the min-max method that is mathematically represented as follows.

(5) $$\begin{array}{c} \vartheta^{{}^{\prime }}=\frac{\vartheta -\vartheta_{min}}{\vartheta_{max}-\vartheta_{min}} \end{array}$$

This normalization technique obtains all the scaled data into the range of 0 to 1. After preprocessing the data, we split the data into two classes, training and testing, where we train 80% of the data and validate 20% of the data. We pass the training dataset ($\Psi_{T1}$) into the ML classifier that classifies the data into attack (1) and nonattack (0) categories.

(6) $$\begin{array}{c} \Psi_{T1}\overset{fed}{\to }{\mathrm{ML}}_{c}\overset{classify}{\to }data\left\{\begin{array}{c} 0\\ 1 \end{array}\right. \end{array}$$

After training the model, we feed the testing dataset ($\Psi_{T2}$) into the ML classifier that validates the results based on the $\Psi_{T1}$.

(7) $$\begin{array}{c} \Psi_{T2}\overset{fed}{\to }{\mathrm{ML}}_{c}\overset{classify}{\to }data\left\{\begin{array}{c} 0\\ 1 \end{array}\right. \end{array}$$

Here, we applied different ML classifiers, such as XGBoost, random forest (RF), k-nearest neighbor (KNN), logistic regression (LR), and support vector machine (SVM), that classify the data into attack and nonattack. Among all these classifiers, we obtained the best results in terms of the accuracy and log loss score from the XGBoost algorithm. The XGBoost classifier gives effective results with the larger dataset. In this classifier, the data are considered for each tree, and we can increase the depth of the tree and decrease the min_child_weight that handles the overfitting issue in the dataset. The classified data are susceptible, which raises the security issue in a public safety application. To overcome this issue, we utilize the IPFS-based blockchain network that offers the data’s authentication and confidentiality, preventing fraud on the dataset.

### 4.3. Blockchain Layer

Blockchain is a decentralized distributed ledger technology that keeps immutable records of transactions. This layer provides security to the classified data by storing them in a shared and transparent ledger that is only accessible to the authorized network members. Here, the data are recorded as a block in a blockchain network through the smart contract ($S_{c}$). Here, a smart contract is an agreement between the contract creator and recipient that executes automatically when all parties can be sure of the conclusion [10]. Then, to provide a reliable connection between contract and storage, there is an interplanetary file system (IPFS) that offers efficient storage and is able to share a large file.

(8) $$\begin{array}{c} data\overset{pass}{\to }S_{c} \end{array}$$

(9) $$\begin{array}{c} S_{c}\overset{Storedata}{\to }I_{f} \end{array}$$

where $I_{f}$ is an IPFS file system that utilizes content-addressing that uniquely retrieves each file in the blockchain network. It stores the data in the blockchain network using a distributed hash table ($H_{T}$). Each stored file is allocated a unique hash value according to the content of the file. The mathematical representation of the aforementioned entity is as follows.

(10) $$\begin{array}{c} I_{f}\overset{generate}{\to }\left\{H_{1},H_{1},\ldots ,H_{n}\right\}\overset{store}{\to }H_{T} \end{array}$$

(11) $$\begin{array}{c} H_{T}\overset{add}{\to }\left\{Block_{1},Block_{2},\ldots ,Block_{n}\right\} \end{array}$$

(12) $$\begin{array}{c} \left\{Block_{1},Block_{2},\ldots ,Block_{n}\right\}\in Blockchain_{n} \end{array}$$

Here, different authorized users such as the fire department, hospital authority, and police retrieve the secure data.

### 4.4. Application Layer

This layer includes different authorities such as the fire department, hospital authority, and police for fire incidents, road accidents, and criminal activity, respectively. This authority uses the classified data for further analysis, prediction, and research purposes. For the proposed architecture, we considered a criminal behavior dataset that has the crime location, crime type, weapon used, weapon type, etc., attributes that classify the data into attack (class 1) or nonattack (class 0). These data will be stored in the IPFS-based blockchain. From that, the police department or any government-regularized authority can access the data securely. The advantage of this layer is that the public safety regulatory body (e.g., emergency response team) acquires the attack-free data. This is because the proposed architecture prohibits any data injection attacks on secure data storage (blockchain); even if the attackers perform any attack on the data storage, it will be known by each blockchain participant due to the transparency features of the blockchain technology. Thus, the proposed architecture offers robust security and reliable communication for IoT-based public safety applications.

### 4.5. Communication Layer

This layer comprises the relation between all four layers. We collected criminal activity data from the data layer that are further passed to the intelligence layer. The data were preprocessed in the intelligence layer and fed into the ML classifier that classifies them into the attack and nonattack classes. The classified data are sensitive to security threats and require immutable and secure storage. For that, we store those data in the decentralized and secure IPFS-based blockchain layer. In the application layer, authorized users, such as employees from the fire department, hospital, and police, access the data using their smartphones, tablet, laptop, or other 6G-based gadgets. They all need a strong communication channel with a high data rate and low latency with secure and relevant data analysis. Moreover, the data layer to the blockchain layer is synchronized using a pipeline via the 6G network interface. Here, the data coming from the blockchain layer are first converted into the digital signal using a Bernoulli binary digital converter and then forwarded into a transmitter block. Here, for the simulation, we considered MATLAB-based 5G toolbox that offers certain parameters, such as frequency range, modulation techniques, channel impairments, sub-carrier spacing, and bandwidth. By modifying these parameters, we can mimic the behavior of 5G interface to 6G interface. We used a frequency range of 75–110 GHz (operational frequency—95 GHz) which is a potential frequency range for beyond 5G specified by the [62], sub-carrier spacing of 240 KHz, and modulation was orthogonal frequency division multiplexing (OFDMA), with polar coding for error detection and correction. The main aim is not to simulate the 6G network for the specified problem formulation but to use essential properties of the 6G network. This is because the 6G simulation is not yet open-sourced by the scientific community; therefore, we have to rely on a 5G simulation setup to impersonate the behavior of the 6G network. Here, the 6G network interface takes the stored data from the blockchain layer and transmits them to the application layer via a 6G network. Thus, data, intelligence, and blockchain layers cumulatively act as a sender that intends to share the data to the application layer via adopting 6G communication for faster and reliable communication.

## 5. Result and Discussion

This section highlightsexperimentation details and results analysis of the proposed architecture in terms of accuracy, log loss score, and validation curve.

### 5.1. Experimental Setup

The proposed architecture utilizes a Google Colaboratory notebook for developing an intelligence layer that uses ML classifiers, Remix integrated development environment (IDE) for the blockchain layer, and MATLAB 2022a for the communication layer. The Google Colaboratory notebook is a web IDE for Python programming that uses various Python libraries such as Pandas, Numpy, Matplotlib, Sklearn, and Scipy for the ML classifiers. To simulate the working of the intelligent layer, we incorporated the Google Colaboratory notebook that uses ML classifiers and provides the classification of the data. Here, different ML classifiers, such as XGboost, RF, KNN, LR, and SVM, have hyperparameters that tailor the classifiers’ behavior and improve performance. Table 3 depicts the parameters used in the proposed architecture. Here, n_estimators, learning_rate, and max_depth are used for the XGBoost classifier, where n_estimators represents the tree’s count. Then, learning_rate is used as the regularization parameter that shows the step size, and the max_depth parameter controls the overfitting in the model. The RF classifier used n_estimators value of 200 and max_depth value of 1. Moreover, KNN uses the n_neighbors parameter to show the total count of neighbors that are utilizedby default for the k-neighbors queries. Furthermore, the uniform weights specify that all points are equally weighted in each neighborhood. The LR classifier uses random_state that shuffles the data and the “lbfgs” solver with max_iter that uses the solvers to converge. For the SVM classifier, we use different parameters such as gamma, probability, and kernel. Here, we use rbf and linear kernel with the kernel coefficient value such as gamma = “auto”, which means 1/n_features.

The classified data have sensitive and confidential information and require secure storage. Therefore, the classified data are stored in the IPFS-based blockchain network using a smart contract in the blockchain layer. The smart contract is implemented using solidity language on the Remix IDE with different functions, such as criminal behaviors() to identify the criminal, weapon() to determine the crime type, addlocation() to know the crime spot, updatecount(), and many more. The smart contract is deployed over the Rinkeby’s network. On a successful compilation and deployment of the smart contract, validated or authenticated data from the smart contract are forwarded to the IPFS-based public blockchain for secure storage. Further, the 6G network is simulated on MATLAB 2022a using a 5G toolbox. This is because there is not yet any open-source software available that provides 6G properties and can integrate that with real-world applications. Due to this constraint, we utilized a 5G-based simulation toolbox, where we changed the simulation parameters, for example, changing the bandwidth allocation, transmit power, sub-carrier spacing (240 KHz), and modulation (OFDMA) techniques of the 5G network to impersonate a 6G network.

### 5.2. Performance Evaluation of the Proposed Architecture

In this subsection, we discuss the results of the ML classifiers, blockchain, and 6G communication. First, we analyze the performance of the different ML classifiers, such as XGBoost, RF, KNN, LR, and SVM, for UAV-based public safety applications such as fire incidents, road accidents, and criminal activity. Here, ML classifiers are used to classify the data into attack means class 1 and nonattack means class 0. We considered the criminal activity dataset that has the crime type, location, weapon type, and criminal behavior records. These records are useful for classifying the arrest and nonarrest categories, which identifies whether the data are vulnerable to attack or not. Here, we fed 80% of the data into the ML classifier that provide training to the classifier. Then, the 20% testing dataset is passed into the ML classifier that validates the training dataset. It also ensures that the trained data will behave consistently and produce the expected results.

**Figure 3:**
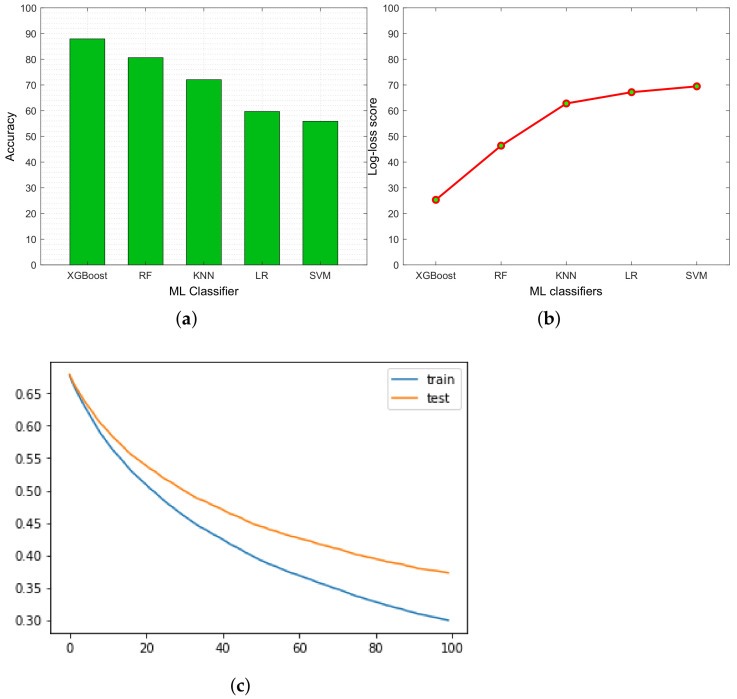
Performance evaluation of ML classifiers. (**a**) Accuracy. (**b**) Log_loss score. (**c**) Validation curve.

Here, Figure 3 depicts the performance of the ML classifiers in terms of the accuracy, log loss score, and validation curve. The accuracy (α) of the ML classifiers represents the ratio of the correctly predicted values out of a total number of predictions. Using an accuracy measure, we know how many times the ML classifier correctly classified the dataset. We can express accuracy in true positive ($T_{\rho }$), false positive ($F_{\rho }$), true negative ($T_{\eta }$), and false negative ($F_{\eta }$) values that are used to represent the number of correct and incorrect predictions of the proposed ML classifier. During the simulation, we observe that a single accuracy parameter is insufficient to remark on the best ML classifier. Therefore, we require additional measures such as precision (β) and recall (γ). Precision represents the correctness of the ML classifier. It determines how good the classifier is at predicting a specific class. It shows the ratio between the true positive samples and all the positive outcomes $T_{\rho }+F_{\rho }$. The recall measure represents the count, which shows how many times the ML classifier was able to determine a specific class. It gives the ratio between true positive samples out of all the cases of true positive and false negative samples $T_{\rho }+F_{\eta }$. Furthermore, harmonic mean of the precision and recall is called F1-score (δ). The mathematical representation of these performance measures are represented as follows:

(13) $$\begin{array}{c} \alpha =\frac{T_{\rho }+T_{\eta }}{T_{\rho }+F_{\rho }+T_{\eta }+F_{\eta }} \end{array}$$

(14) $$\begin{array}{c} \beta =\frac{T_{\rho }}{T_{\rho }+F_{\rho }} \end{array}$$

(15) $$\begin{array}{c} \gamma =\frac{T_{\rho }}{T_{\rho }+F_{\eta }} \end{array}$$

(16) $$\begin{array}{c} \delta =2*\frac{\beta *\gamma }{\beta +\gamma } \end{array}$$

Figure 3a illustrates the accuracy of the different ML classifiers such as XGBoost, RF, KNN, LR, and SVM. From the graph, we found that XGBoost classifier gives better accuracy (87.93%) compared to the other classifiers because it handles sparse data and executes parallel computing. On the contrary, SVM did not perform well because it required high training time for the large dataset. Table 4 shows that the XGBoost classifier outperforms any other ML classifiers in terms of accuracy, precision, recall, and F1-score.

To further measure the ML classifier’s performance, we used a log loss score that indicates how close the prediction probability is to the corresponding actual value in the binary classification case. It represents the probabilities of the correct class label, which means that if the predicted probability is high, it will obtain a low log loss core and vice versa. Figure 3b shows the log loss score of the different ML classifiers that evaluate the performance of the classifiers. From the empirical results, we analyzed that there is a higher divergence in the log loss score of the LR and SVM classifiers. Here, XGBoost’s log loss score is small; therefore, it performed well compared to the other ML classifiers. Additionally, Figure 3c depicts the validation curve of the XGBoost classifier that indicates the sensitivity between changes in a classifier’s accuracy and changes in some parameters of the classifier. Here, if the training score is more than the validation score, the estimator is overfitting, and otherwise performs well.

**Figure 4:**
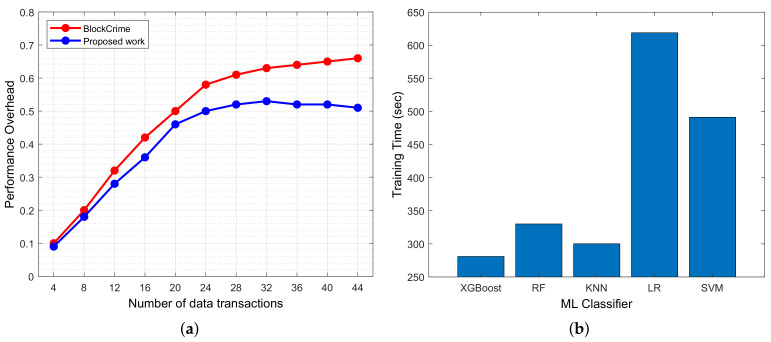
(**a**) Comparison of performance overhead between [63] and proposed work. (**b**) ML classifier training time comparison.

Moreover, we want to mention that most of the literature we observed does not amalgamate the mentioned key enabler technologies, i.e., blockchain, 6G network, AI, etc. Their approach is based on a single technology, but by using that, there must be a loophole that exists that the attackers can exploit. Therefore, an amalgamation of technology is needed to offer a secure pipeline for data exchange for critical applications, such as public safety applications. The work of [63] is similar to our proposed work with better accuracies; however, their work is based on an image dataset where they used rigorous data augmentation techniques and deep learning approaches to acquire better accuracies. Because of data augmentation and deep learning approaches, their proposed model is highly complex and does not have a commendable system response time. This is because deep learning techniques have a higher training time, due to which the response becomes higher; hence, one could not send critical data on time to the application layer. This can also be seen in Figure 4a, where performance overhead is compared between [63] and the proposed work. It is clear from the graph that due to the high training time, the work of [63] has a high-performance overhead compared to our work. Contrary, the proposed work uses text data where minimal data preprocessing steps are required to train ML classifiers. This can be seen in Figure 4b, where training time is computed for all ML classifiers. XGBoost is outperforming other classifiers by attaining a training time of 280.97 s. In [63], the authors used 1400 iterations to obtain a better training accuracy, which takes longer training time compared to our work.

**Figure 5:**
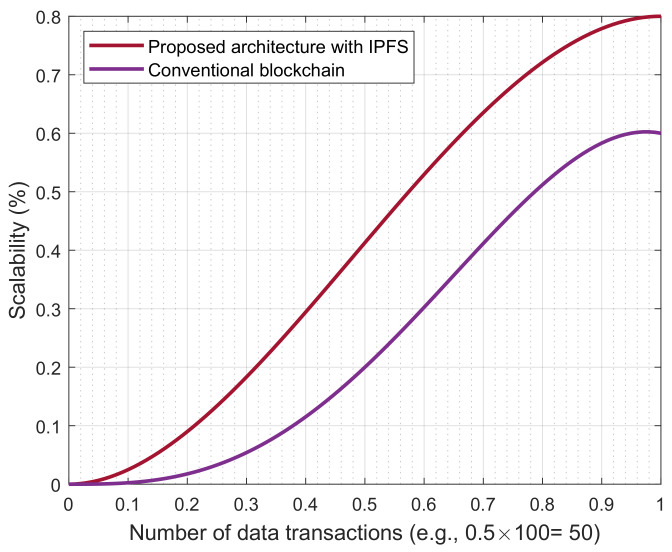
Comparison of blockchain scalability.

Figure 5 illustrates the blockchain scalability comparison between the proposed blockchain with IPFS and the traditional blockchain. Generally, an IPFS-based blockchain is a decentralized protocol and is open to the public; therefore, anyone who has content hash can access the network. Moreover, it offers persistent data storage, fault tolerance against a single point of failure, and a censorship resistance mechanism. Due to the aforesaid benefits, the IPFS-based blockchain has a better response time than the traditional blockchain (i.e., not using IPFS). In addition, the immutable nature of the IPFS-based blockchain eases numerous cybersecurity threats. It has no overheads of the consensus protocols to add the data and only stores the content hash in the network. Thus, the consensus and data aggregation with transformation must be applied before submitting a block or file to IPFS storage. Due to this mechanism, the IPFS-based blockchain is faster and more scalable than the conventional blockchain network.

**Figure 6:**
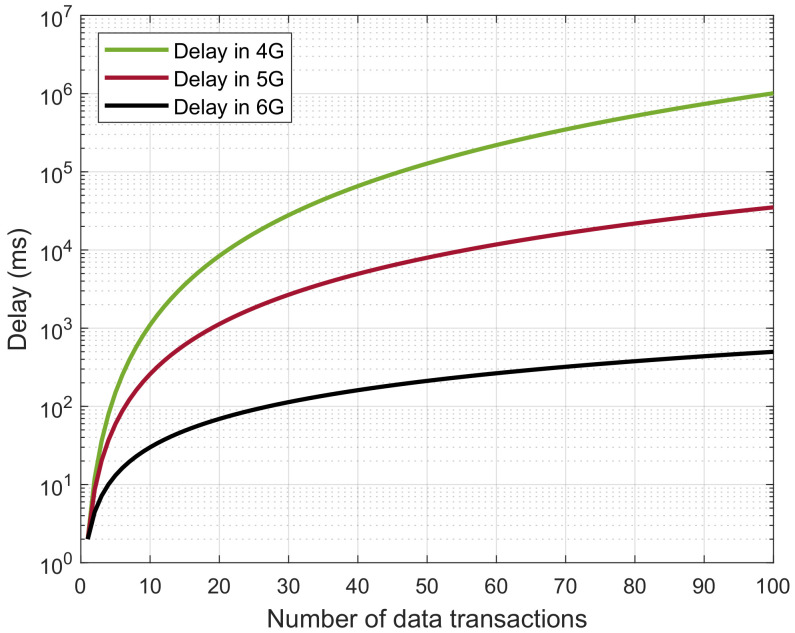
Comparison of latency with traditional network interface.

Furthermore, the proposed architecture consists of UAVs for data accumulation and dissemination between different participants of public safety applications. As public safety applications are categorized under the umbrella of mission-critical applications, they must ensure reliable and low-latency communication among all the entities of the proposed architecture. Here, 6G networks fulfill the requirements essential to the proposed architecture. The 6G network offers high data-transmission rates, with ultra-low latency that makes it 1000 times faster than 5G networks. Therefore, the network’s transmitter and receiver have the minimum packet loss ratio. Figure 6 shows the packet drop ratio in the 4G, 5G, and 6G networks.

## 6. Challenges of Adoption of Blockchain in 6G Network

**Figure 7:**
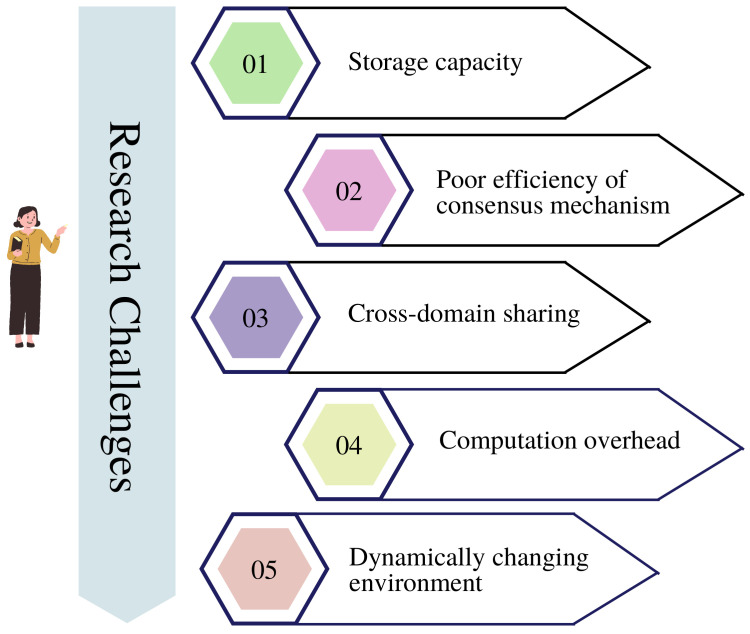
Blockchain adoption challenges in 6G network.

Blockchain technology entrenches data security, user identity, information authenticity, and privacy mechanisms into the 6G communication system. However, poor efficiency of consensus mechanism, computation overhead of network systems, storage capacity, limited network and computing resources, and cross-network sharing imposes a new challenge in blockchain-based 6G application. This section provides the possible challenges of adopting blockchain in the 6G network. Figure 7 displays the research challenges when adopting blockchain in the 6G network.

### 6.1. Storage Capacity

Blockchain offers decentralized architecture with an immutable ledger that securely stores the data transaction that cannot be tampered or altered. Blockchain permanently keeps data in immutable blocks and is not allowed to be altered or deleted by anyone; therefore, it keeps increasing data volume and raising data storage capacity issues. During peak time, an increasing number of users and data transactions flood the network, leading to transaction delays and network overloading issues. Moreover, due to a growing demand for data storage capacity and bandwidth, nodes cannot synchronize with each other to join the network [64]. Various cloud platforms exist, such as IPFS, Merkle tree-based file systems (MTFS), StorJ, swarm, etc., that resolve the data storage issue in the blockchain-based 6G network. The research community has adopted these data storage platforms with blockchain and proposed solutions in supply chain management, smart agriculture, smart city, etc., to resolve storage issues. For example, Pawar et al. [65] proposed an IPFS-enabled blockchain repository for supply chain management to minimize file upload and download time. Kumar et al. [66] introduced a secured privacy-preserving scheme to maintain scalability using IPFS-based off-chain storage for smart agricultural UAVs data. However, these solutions face reliability, privacy, and security issues, such as that IPFS does not provide private file storage; it only offers public file access. Likewise, MTFS has access speed and scalability problems with public file systems. From that, it has been noticed that the limited data storage capacity of blockchain is a notable challenge in the current and future networks.

### 6.2. Poor Efficiency of Consensus Mechanism

The blockchain network depends on various consensus mechanisms such as practical Byzantine fault tolerance (PBFT), proof-of-work, and proof-of-stake for enterprise and permissionless blockchains. However, these algorithms are inadequate because they consume more time and energy for block production. Researchers have designed various consensus algorithms to monitor and verify block production in 6G network. For example, Wan et al. [67] and Zhang et al. [68] proposed a consensus-algorithm-based strategy for collision avoidance in UAV swarms and fixed-wing UAVs, respectively. Still, there is scope for research in this field because the algorithms require higher energy consumption and are vulnerable to malicious attacks such as the 51% attack, Sybil attack, race attack, and Finney attack.

### 6.3. Cross-Network or Cross-Domain Sharing

Blockchain introduces an interoperability feature that offers a cross-chain mechanism to transmit asserts, transaction values, virtual contracts, and data between two or more blockchain networks. Unfortunately, the lack of adequate privacy and maintaining the same level of security raise cross-network sharing issues in blockchain-based 6G applications. Recently, much work has been carried out for cross-network sharing in blockchain-enabled smart applications. For example, in [69], the authors proposed a cross-network data-sharing mechanism for the smart grid. Likewise, Xue et al. [70] presented a cross-domain authentication scheme for medical information sharing. Besides these advantages, several challenges remain to be addressed, such as data ownership protection with QoS-based network heterogeneity.

### 6.4. Computation Overhead

The 6G applications are bandwidth- and resource-hungry; they require real-time data transmission. However, the increasing number of network users hinders the QoS of the developed applications. Therefore, blockchain integrates with 6G technologies, reducing the complex computational cost. A lot of research has been carried out to overcome the computation overhead issue in blockchain-based 6G applications. For instance, the work of Chai et al. [71] offered a diffused-PBFT approach to reduce computation overhead and consensus latency. Likewise, in [72], the authors proposed an LSTM-based scheme to optimize the computation offloading in a satellite-UAV-based IoT system. Then, Ahmed et al. [73] introduced blockchain in VANET for vehicle authentication and emergency message transmission between the Internet of vehicles (IoVs). They encrypted blockchain servers using RSA-1024, a digital signature algorithm, to minimize the computation overhead. However, each participating block of the blockchain and consensus algorithms, such as proof-of-work and PBFT, require more computational power for calculation. On top of that, the continuous growth of IoT devices and smart applications leads to high congestion in the network and computation overhead, where it is infeasible to properly apply a blockchain-based consensus approach.

### 6.5. Dynamically Changing Environment

Digital technologies such as AI, IoT, and robotics are continuously growing and changing the future perspective, industrial demand, and human behavior. These technologies are incorporated into various smart applications such as e-healthcare, smart homes, smart agriculture, holographic communications and many more. According to user demand for a high transmission rate with lower latency, these technologies should respond in the real-time environment of industries and organizations. Moreover, blockchain networks are incorporated in dynamic, experimental, and sustainable future technologies to introduce trust, authenticity, security, and privacy [74]. The research community has offered many solutions based on blockchain for a dynamic changing environment. For example, Xiao et al. [75] proposed temporal pattern attention and enabled the LSTM approach to sense the change of natural gas deliverability in natural-gas-based IoT systems. They utilized a blockchain-based smart contract that dynamically matched the purchase and sale details of the gas; it also enhanced the buyer’s and the seller’s interests. As a result, they could predict the outcome of natural gas in real-time according to the dynamic demand for sales. However, modeling such a real-time technique is highly complex and time-consuming in a dynamic changing environment.

## 7. Conclusions

Blockchain is distributed ledger technology that offers reliability, security, immutability, and privacy in the 6G-based futuristic smart application. It proves its effectiveness by providing trust been two parties in the wireless communication network. Many research communities have discussed the adoption of blockchain for 6G-based smart applications with new digital technologies, such as IoT, AI, and MEC. This technology offers real-time communication with a higher transmission rate; however, it adds various challenges, making it difficult to adapt to a 6G network. Therefore, it is required to propose an exhaustive work highlighting the findings of multiple researchers who worked in the blockchain-based 6G networks. We discussed various 6G applications where blockchain is adopted for the security and privacy of data. Further, we discussed blockchain-based services in the 6G network. Then, we proposed layer-wise architecture by adopting AI, blockchain, and a 6G network to protect sensitive crime data in public safety applications. An attacker tries to manipulate criminal activity information systems to avoid legal action; therefore, the proposed architecture offers a secure pipeline to protect public safety applications from the nefarious offense. The proposed architecture is evaluated on numerous performance evaluation metrics such as accuracy, scalability, and packet drop rate. Lastly, we identified the challenge of adopting the blockchain in the 6G wireless networks.

In the future, based on this work, we will develop a novel solution that mitigates the identified issues and improves the performance of the blockchain-based 6G system.

## Figures and Tables

**Table 1:** A comparative analysis of proposed work with the existing studies.

Author	Year	Objective	6G	Security	Approach	Pros	Cons
Xu et al. [33]	2019	Smart public safety system.	✗	Blockchain.	BlendMAS.	The scheme offers secure and smart data sharing with access control.	Does not yet explore 6G networks.
Ihinosen et al. [32]	2020	Safety and security in rideshare service.	✗	Using facial recognition API.	GPS tracking and 2-way rating system.	Developed mobile ridesharing scheme enhance the security	Did not consider latency and delay.
Fitwi et al. [34]	2020	Video surveillance systems security.	✗	Blockchain.	Lib-Pri scheme.	The scheme offers real-time video analytics by identifying fugitives using facial features.	Did not consider data confidentiality and scalability.
Makhdoom et al. [35]	2021	To prevent cyberattack incidents.	✗	Blockchain.	Blockchain-enabled collaborative intrusion detection systems.	The scheme enabled node and application authorization.	Does not discuss the scalability of the system.
Wu et al. [36]	2022	On-site safety for tower cranes.	✗	Blockchain.	Smart contract, consensus, and Hyperledger-Fabric-based framework.	The scheme enhanced the safety performance.	Did not consider latency, throughput, and computation overhead analysis.
Yu et al. [37]	2022	Security and privacy of IIoT data.	✗	Blockchain and cryptography.	STCChain.	The scheme prevented data stealing and attacks.	Does not incorporate 6G-based solution.
Na et al. [38]	2022	Crime and accident prevention for autonomous vehicles.	✗	Blockchain.	Multisignature-enabled access control using GPS data.	The scheme offers privacy and security of the image and video data with lower latency.	The scheme limits verifying the reliability of GPS data.
Proposed scheme	2022	Public safety to monitor criminal activity.	✓	Blockchain.	Layered architecture incorporating blockchain and 6G to detect crime.	The scheme uses different ML classifier to identify the attack and nonattack from the criminal activity dataset.	-

**Table 3:** Parameters of the proposed architecture.

Hyperparameters Used by ML Classifiers	
**ML Classifiers**	**Parameters Used**
XGBoost	n_estimators: 100, learning_rate: 0.1, max_depth: 1
RF	n_estimators: 200, max_depth: 5
KNN	n_neighbors: 5, weights: ‘uniform’
LR	random_state:1, solver: ‘lbfgs’, max_iter: 100
SVM	gamma: ‘auto’, probability: True, kernel: [‘rbf’,‘linear’]
Communication Layer Parameters	
Frequency range	75–110 GHz
Sub-carrier spacing	240 KHz
Channel coding	Polar coding
Channel fading	com.Rayleigh fading channel
Modulation	OFDMA
Blockchain Layer Parameters	
Compiler language	Solidity
Network	Rinkeby’s
Environment	Remix VM

**Table 4:** Comparison of ML classifiers’ performance for different metrics.

	Accuracy (%)	Precision (%)	Recall (%)	F1 Score (%)
XGBoost	87.93	85.45	80.32	83.23
RF	80.63	79.34	76.21	74.43
KNN	72.06	71.21	69.32	70.21
LR	59.68	55.43	51.22	52.10
SVM	55.87	52.32	50.32	51.21

## Data Availability

No data are associated with this research work.

## References

[1] Raja G., Sai S.G., Rajakumar B.R., Gurumoorthy S., Dev K., Magarini M. Nexus of 6G and Blockchain for Authentication of Aerial and IoT Devices. Proceedings of the ICC 2022—IEEE International Conference on Communications.

[2] Gupta R., Reebadiya D., Tanwar S. (2021). 6G-enabled Edge Intelligence for Ultra—Reliable Low Latency Applications: Vision and Mission. Comput. Stand. Interfaces.

[3] Sekaran R., Patan R., Raveendran A., Al-Turjman F., Ramachandran M., Mostarda L. (2020). Survival Study on Blockchain Based 6G-Enabled Mobile Edge Computation for IoT Automation. IEEE Access.

[4] Singh R., Tanwar S., Sharma T.P. (2020). Utilization of blockchain for mitigating the distributed denial of service attacks. Secur. Priv..

[5] Velliangiri S., Manoharan R., Ramachandran S., Rajasekar V. (2022). Blockchain Based Privacy Preserving Framework for Emerging 6G Wireless Communications. IEEE Trans. Ind. Informatics.

[6] Bindu G., Iwin Thanakumar J.S., Kanakala V.R., Niharika G.L.K., Raj B.E. Impact of Blockchain Technology in 6G Network: A Comprehensive survey. Proceedings of the 2022 International Conference on Inventive Computation Technologies (ICICT).

[7] Senkyire I.B., Kester Q.A. Validation of Forensic Crime Scene Images Using Watermarking and Cryptographic Blockchain. Proceedings of the 2019 International Conference on Computer, Data Science and Applications (ICDSA).

[8] Gupta R., Shukla A., Tanwar S. (2021). BATS: A Blockchain and AI-Empowered Drone-Assisted Telesurgery System Towards 6G. IEEE Trans. Netw. Sci. Eng..

[9] Patel K., Mehta D., Mistry C., Gupta R., Tanwar S., Kumar N., Alazab M. (2020). Facial Sentiment Analysis Using AI Techniques: State-of-the-Art, Taxonomies, and Challenges. IEEE Access.

[10] Gupta R., Shukla A., Tanwar S. AaYusH: A Smart Contract-Based Telesurgery System for Healthcare 4.0. Proceedings of the 2020 IEEE International Conference on Communications Workshops (ICC Workshops).

[11] Pradhan N.R., Rout S.S., Singh A.P. Blockchain Based Smart Healthcare System for Chronic—Illness Patient Monitoring. Proceedings of the 2020 3rd International Conference on Energy, Power and Environment: Towards Clean Energy Technologies.

[12] Wang S., Wang J., Wang X., Qiu T., Yuan Y., Ouyang L., Guo Y., Wang F.Y. (2018). Blockchain-Powered Parallel Healthcare Systems Based on the ACP Approach. IEEE Trans. Comput. Soc. Syst..

[13] Leng J., Ye S., Zhou M., Zhao J.L., Liu Q., Guo W., Cao W., Fu L. (2021). Blockchain-Secured Smart Manufacturing in Industry 4.0: A Survey. IEEE Trans. Syst. Man Cybern. Syst..

[14] Leng J., Yan D., Liu Q., Xu K., Zhao J.L., Shi R., Wei L., Zhang D., Chen X. (2020). ManuChain: Combining Permissioned Blockchain With a Holistic Optimization Model as Bi-Level Intelligence for Smart Manufacturing. IEEE Trans. Syst. Man Cybern. Syst..

[15] Lee C.K.M., Huo Y.Z., Zhang S.Z., Ng K.K.H. (2020). Design of a Smart Manufacturing System With the Application of Multi-Access Edge Computing and Blockchain Technology. IEEE Access.

[16] Teng Y., Li L., Song L., Yu F.R., Leung V.C.M. (2022). Profit Maximizing Smart Manufacturing Over AI-Enabled Configurable Blockchains. IEEE Internet Things J..

[17] Khalil U., Mueen-Uddin, Malik O.A., Hussain S. (2022). A Blockchain Footprint for Authentication of IoT-Enabled Smart Devices in Smart Cities: State-of-the-Art Advancements, Challenges and Future Research Directions. IEEE Access.

[18] Bai Y., Hu Q., Seo S.H., Kang K., Lee J.J. (2022). Public Participation Consortium Blockchain for Smart City Governance. IEEE Internet Things J..

[19] Kumar P., Kumar R., Srivastava G., Gupta G.P., Tripathi R., Gadekallu T.R., Xiong N.N. (2021). PPSF: A Privacy-Preserving and Secure Framework Using Blockchain-Based Machine-Learning for IoT-Driven Smart Cities. IEEE Trans. Netw. Sci. Eng..

[20] Rahman M.A., Rashid M.M., Hossain M.S., Hassanain E., Alhamid M.F., Guizani M. (2019). Blockchain and IoT-Based Cognitive Edge Framework for Sharing Economy Services in a Smart City. IEEE Access.

[21] Chattaraj D., Bera B., Das A.K., Saha S., Lorenz P., Park Y. (2021). Block-CLAP: Blockchain-Assisted Certificateless Key Agreement Protocol for Internet of Vehicles in Smart Transportation. IEEE Trans. Veh. Technol..

[22] Xia S., Lin F., Chen Z., Tang C., Ma Y., Yu X. (2020). A Bayesian Game Based Vehicle-to-Vehicle Electricity Trading Scheme for Blockchain-Enabled Internet of Vehicles. IEEE Trans. Veh. Technol..

[23] Ghourab E.M., Azab M., Ezzeldin N. (2022). Blockchain-Guided Dynamic Best-Relay Selection for Trustworthy Vehicular Communication. IEEE Trans. Intell. Transp. Syst..

[24] Liang X., An N., Li D., Zhang Q., Wang R. A Blockchain and ABAC Based Data Access Control Scheme in Smart Grid. Proceedings of the 2022 International Conference on Blockchain Technology and Information Security (ICBCTIS).

[25] Samy S., Azab M., Rizk M. Towards a Secured Blockchain-based Smart Grid. Proceedings of the 2021 IEEE 11th Annual Computing and Communication Workshop and Conference (CCWC).

[26] Wang Y., Su Z., Zhang N., Chen J., Sun X., Ye Z., Zhou Z. (2021). SPDS: A Secure and Auditable Private Data Sharing Scheme for Smart Grid Based on Blockchain. IEEE Trans. Ind. Informatics.

[27] Hao X., Ren W., Choo K.K.R., Xiong N.N. (2022). A Self-Trading and Authenticated Roaming Scheme Based on Blockchain for Smart Grids. IEEE Trans. Ind. Informatics.

[28] Zhang F., Zhang Y., Lu W., Gao Y., Gong Y., Cao J. (2022). 6G-Enabled Smart Agriculture: A Review and Prospect. Electronics.

[29] Tran Q.N., Turnbull B.P., Wu H.T., de Silva A.J.S., Kormusheva K., Hu J. (2021). A Survey on Privacy-Preserving Blockchain Systems (PPBS) and a Novel PPBS-Based Framework for Smart Agriculture. IEEE Open J. Comput. Soc..

[30] Vangala A., Sutrala A.K., Das A.K., Jo M. (2021). Smart Contract-Based Blockchain-Envisioned Authentication Scheme for Smart Farming. IEEE Internet Things J..

[31] Yang X., Li M., Yu H., Wang M., Xu D., Sun C. (2021). A Trusted Blockchain-Based Traceability System for Fruit and Vegetable Agricultural Products. IEEE Access.

[32] Ihinosen A.B., Mhlanga S.T., Lall M. Enhancing safety and security in a dynamic rideshare service. Proceedings of the 2020 5th International Conference on Computing, Communication and Security (ICCCS).

[33] Xu R., Nikouei S.Y., Chen Y., Blasch E., Aved A. BlendMAS: A Blockchain-Enabled Decentralized Microservices Architecture for Smart Public Safety. Proceedings of the 2019 IEEE International Conference on Blockchain (Blockchain).

[34] Fitwi A., Chen Y., Zhu S. A Lightweight Blockchain-Based Privacy Protection for Smart Surveillance at the Edge. Proceedings of the 2019 IEEE International Conference on Blockchain (Blockchain).

[35] Makhdoom I., Hayawi K., Kaosar M., Mathew S.S., Masud M.M. Blockchain-based Secure CIDS Operation. Proceedings of the 2021 5th Cyber Security in Networking Conference (CSNet).

[36] Wu H., Zhong B., Li H., Chi H.L., Wang Y. (2022). On-site safety inspection of tower cranes: A blockchain-enabled conceptual framework. Saf. Sci..

[37] Yu K., Tan L., Yang C., Choo K.K.R., Bashir A.K., Rodrigues J.J.P.C., Sato T. (2022). A Blockchain-Based Shamir’s Threshold Cryptography Scheme for Data Protection in Industrial Internet of Things Settings. IEEE Internet Things J..

[38] Na D., Park S. (2022). Blockchain-Based Dashcam Video Management Method for Data Sharing and Integrity in V2V Network. IEEE Access.

[39] Abdulqadder I.H., Zhou S. (2022). SliceBlock: Context-Aware Authentication Handover and Secure Network Slicing Using DAG-Blockchain in Edge-Assisted SDN/NFV-6G Environment. IEEE Internet Things J..

[40] He G., Su W., Gao S., Liu N., Das S.K. (2022). NetChain: A Blockchain-Enabled Privacy-Preserving Multi-Domain Network Slice Orchestration Architecture. IEEE Trans. Netw. Serv. Manag..

[41] Chen F., Li Z., Li B., Deng C., Tian Z., Lin N., Wan Y., Bao B. Blockchain-based Optical Network Slice Rental Approach for IoT. Proceedings of the 2020 IEEE Computing, Communications and IoT Applications (ComComAp).

[42] Vora J., Kaneriya S., Tanwar S., Tyagi S., Kumar N., Obaidat M. (2019). TILAA: Tactile Internet-based Ambient Assistant Living in fog environment. Future Gener. Comput. Syst..

[43] Liu X., Lam K.Y., Li F., Zhao J., Wang L., Durrani T.S. (2021). Spectrum Sharing for 6G Integrated Satellite-Terrestrial Communication Networks Based on NOMA and CR. IEEE Netw..

[44] Tyagi S., Tanwar S., Gupta S.K., Kumar N., Rodrigues J.J. (2015). A lifetime extended multi-levels heterogeneous routing protocol for wireless sensor networks. Telecommun. Syst..

[45] Saha R.K. (2020). Approaches to Improve Millimeter-Wave Spectrum Utilization Using Indoor Small Cells in Multi-Operator Environments Toward 6G. IEEE Access.

[46] Lu W., Si P., Huang G., Han H., Qian L., Zhao N., Gong Y. (2021). SWIPT Cooperative Spectrum Sharing for 6G-Enabled Cognitive IoT Network. IEEE Internet Things J..

[47] Gupta R., Kumari A., Tanwar S. (2021). Fusion of blockchain and artificial intelligence for secure drone networking underlying 5G communications. Trans. Emerg. Telecommun. Technol..

[48] Liu L., Liang W., Mang G., Dong Z. Blockchain Based Spectrum Sharing over 6G Hybrid Cloud. Proceedings of the 2021 International Wireless Communications and Mobile Computing (IWCMC).

[49] Zhang H., Leng S., Wu F., Chai H. (2022). A DAG Blockchain-Enhanced User-Autonomy Spectrum Sharing Framework for 6G-Enabled IoT. IEEE Internet Things J..

[50] Manogaran G., Rawal B.S., Saravanan V., Kumar P.M., Martínez O.S., Crespo R.G., Montenegro-Marin C.E., Krishnamoorthy S. (2020). Blockchain based integrated security measure for reliable service delegation in 6G communication environment. Comput. Commun..

[51] Khowaja S.A., Khuwaja P., Dev K., Lee I.H., Khan W., Wang W., Qureshi N.M.F., Magarini M. (2022). A secure data sharing scheme in Community Segmented Vehicular Social Networks for 6G. IEEE Trans. Ind. Inform..

[52] Zhang F., Guo S., Qiu X., Xu S., Qi F., Wang Z. (2021). Federated Learning Meets Blockchain: State Channel based Distributed Data Sharing Trust Supervision Mechanism. IEEE Internet Things J..

[53] Li H., Pei L., Liao D., Chen S., Zhang M., Xu D. (2020). FADB: A Fine-Grained Access Control Scheme for VANET Data Based on Blockchain. IEEE Access.

[54] Cao H., Du J., Zhao H., Luo D.X., Kumar N., Yang L., Yu F.R. (2022). Toward Tailored Resource Allocation of Slices in 6G Networks With Softwarization and Virtualization. IEEE Internet Things J..

[55] Sadi Y., Erkucuk S., Panayirci E. Flexible Physical Layer based Resource Allocation for Machine Type Communications Towards 6G. Proceedings of the 2020 2nd 6G Wireless Summit (6G SUMMIT).

[56] Dai H., Zhang C., Luo J., Li C., Wang B. QoE- Driven Resource Allocation for Secure URLLC in 6G-Enabled IoT Networks. Proceedings of the 2021 13th International Conference on Wireless Communications and Signal Processing (WCSP).

[57] Lin K., Li Y., Zhang Q., Fortino G. (2021). AI-Driven Collaborative Resource Allocation for Task Execution in 6G-Enabled Massive IoT. IEEE Internet Things J..

[58] Li M., Pei P., Yu F.R., Si P., Li Y., Sun E., Zhang Y. (2022). Cloud-Edge Collaborative Resource Allocation for Blockchain-Enabled Internet of Things: A Collective Reinforcement Learning Approach. IEEE Internet Things J..

[59] Jain D.K., Tyagi S.K.S., Neelakandan S., Prakash M., Natrayan L. (2022). Metaheuristic Optimization-Based Resource Allocation Technique for Cybertwin-Driven 6G on IoE Environment. IEEE Trans. Ind. Inform..

[60] Yang L., Li M., Si P., Yang R., Sun E., Zhang Y. (2021). Energy-Efficient Resource Allocation for Blockchain-Enabled Industrial Internet of Things With Deep Reinforcement Learning. IEEE Internet Things J..

[61] Crimes in Chicago. https://www.kaggle.com/datasets/currie32/crimes-in-chicago.

[62] ConsenSys Fundamentals of THz Technology for 6G. https://www.rohde-schwarz.com/in/solutions/test-and-measurement/wireless-communication/cellular-standards/6g/white-paper-fundamentals-of-thz-technology-for-6g-by-rohde-schwarz-registration-255934.html.

[63] Patel D., Sanghvi H., Jadav N.K., Gupta R., Tanwar S., Florea B.C., Taralunga D.D., Altameem A., Altameem T., Sharma R. (2022). BlockCrime: Blockchain and Deep Learning-Based Collaborative Intelligence Framework to Detect Malicious Activities for Public Safety. Mathematics.

[64] Arigela S.S.D., Voola P. Detecting and Identifying Storage issues using Blockchain Technology. Proceedings of the 2022 International Conference on Advances in Computing, Communication and Applied Informatics (ACCAI).

[65] Pawar M.K., Patil P., Sharma M., Chalageri M. Secure and Scalable Decentralized Supply Chain Management Using Ethereum and IPFS Platform. Proceedings of the 2021 International Conference on Intelligent Technologies (CONIT).

[66] Kumar R., Kumar P., Tripathi R., Gupta G.P., Gadekallu T.R., Srivastava G. (2021). SP2F: A secured privacy-preserving framework for smart agricultural Unmanned Aerial Vehicles. Comput. Netw..

[67] Wan Y., Tang J., Lao S. (2019). Distributed Conflict-Detection and Resolution Algorithm for UAV Swarms Based on Consensus Algorithm and Strategy Coordination. IEEE Access.

[68] Zhang J., Yan J., Zhang P., Kong X. (2018). Collision Avoidance in Fixed-Wing UAV Formation Flight Based on a Consensus Control Algorithm. IEEE Access.

[69] Hao Y., Cao H., Wang Y., Xiong L., Liu X., Yang L., Ni J. Blockchain-Enabled Secure and Transparent Cross-Regional Model Updating and Sharing Approach in Smart Grid. Proceedings of the 2021 IEEE 10th Data Driven Control and Learning Systems Conference (DDCLS).

[70] Xue L., Huang H., Xiao F., Wang W. (2022). A Cross-domain Authentication Scheme Based on Cooperative Blockchains Functioning with Revocation for Medical Consortiums. IEEE Trans. Netw. Serv. Manag..

[71] Chai H., Leng S., He J., Zhang K., Cheng B. (2022). CyberChain: Cybertwin Empowered Blockchain for Lightweight and Privacy-Preserving Authentication in Internet of Vehicles. IEEE Trans. Veh. Technol..

[72] Mao B., Tang F., Kawamoto Y., Kato N. (2021). Optimizing Computation Offloading in Satellite-UAV-Served 6G IoT: A Deep Learning Approach. IEEE Netw..

[73] Ahmed M., Moustafa N., Akhter A.F.M.S., Razzak I., Surid E., Anwar A., Shah A.F.M.S., Zengin A. (2022). A Blockchain-Based Emergency Message Transmission Protocol for Cooperative VANET. IEEE Trans. Intell. Transp. Syst..

[74] Muhs J. How Does Blockchain Technology Help Deliver the Future…Now_?. https://economictimes.indiatimes.com/small-biz/security-tech/technology/how-does-blockchain-technology-help-deliver-the-futurenow/articleshow/86313876.

[75] Xiao W., Liu C., Wang H., Zhou M., Hossain M.S., Alrashoud M., Muhammad G. (2021). Blockchain for Secure-GaS: Blockchain-Powered Secure Natural Gas IoT System with AI-Enabled Gas Prediction and Transaction in Smart City. IEEE Internet Things J..

